# The Cause and Treatment of Septic Peritonitis

**Published:** 1898-12

**Authors:** 


### Selections and Abstracts.

### THE CAUSE AND TREATMENT OF SEPTIC PERITONITIS.

Septic peritonitis ranks as one of the most important of surgical conditions, both on account of its frequency and its fatality. For this reason it is important to review our knowledge of the subject from time to time.

First, as to the cause of this affection. A large amount of literature bearing upon this point now exists. The most recent investigations tend to show that all forms of peritonitis are the result of contamination with micro-organisms or their products. The cases heretofore termed "idiopathic" were so called because the source of the inflammation was not apparent. In the "traumatic" variety, the injury doubtless does nothing more than to establish a focus of lessened resistance, which permits the germs that reach this situation, either through the blood or otherwise, to develop. The "septic" and "suppurative" forms are, of course, of bacterial origin.

Among the micro-organisms that have been found associated with peritoneal inflammations by bacteriological examination may be mentioned the colon bacillus, streptococcus, staphylococcus pyogenes albus, aureus, and citreus, pneumococcus, and bacillus pyocyaneous. It is still uncertain whether the typhoid bacillus can cause peritonitis, and it is claimed that the gonococcus of itself is incapable of exciting inflammation of the peritoneum. The list above given can not be considered exhaustive in any sense, but it represents the more common, well-established, and generally accepted sources of the affection.

Of all the forms of peritonitis, that due to streptococcus infection is probably the most virulent, although there are cases, apparently due to the colon bacillus, that are almost, if not quite, as severe in type. The mildest form of peritoneal infection is that due to the staphylococcus. It must be borne in mind, however, that the virulence of any given infection depends upon other factors than the particular variety of germ from which it originated. These unfortunately are not yet understood. In addition, there is very frequently a mixed infection present, which, of course, would modify the conditions present in many important particulars. The additional fact must be borne in mind that, in a case of mixed peritoneal infection, the germ that caused the inflammation may rapidly disappear, either on account of feeble resistance or by being destroyed by the accompanying micro-organism, the latter having greater resistance and more actively growing properties.

The commoner causes of bacterial infection of the peritoneum are rupture or perforation of the hollow viscera, penetrating wounds of the abdominal wall, including operations, and infection conveyed through the blood. It is a matter of common observation that peritonitis does not follow invariably upon exposure of the peritoneum to septic matter. This membrane is therefore capable, under favorable conditions, of disposing of a certain amount of purulent matter, and even bacteria, as well as fluids. This absorptive function has been made the subject of careful study by a number of investigators. Among the results of this work may be mentioned the discovery of stomata or open lymph-spaces, situated between the connective-tissue fibres of the peritoneum covering the central tendon of the diaphragm. It is at this point that the most rapid absorption takes place; solid particles being thus removed from the abdominal cavity as well as fluids. It is claimed that there is a current within the abdomen which tends to carry foreign substances from the pelvis towards the diaphragm, regardless of the posture of the individual. It has been shown experimentally that in peritonitis the lymph-spaces become filled with masses of bacteria and leucocytes, and as the result of mechanical obstruction and inflammatory action the power of further absorption is completely arrested. The products of bacterial growth, therefore, remain in the peritoneal cavity, and more or less rapidly poison the patient, according to the virulence of the particular case. As Treves and others have pointed out, death from peritonitis is the result of toxemia, and not of the inflammation *per se*. These facts would appear to demonstrate conclusively the value of establishing drainage as early as possible in all cases of septic peritonitis.

With this theoretical deduction clinical experience is in accord. A large number of cases of septic peritonitis is now on record in which life has undoubtedly been saved by incision and drainage. The latest statistics show that between 20 and 40 per cent. of recoveries may be expected from these operations. The results obtained appear to be improving each year, as the patients are operated upon earlier, and as the methods improve.

The technique at present accepted as the best consists of a large median incision, with a lateral incision, if needed, to give better access to any particular focus of disease, copious flushing of the cavity with normal salt solution at a temperature of 110° to 114° F., and the establishment of free drainage. If the intestines are covered with lymph, it is recommended to cleanse them by sponging with sterile gauze pads.

For this purpose it may be necessary to deliver the small intestine through the abdominal incision. During the cleansing process, the bowels should be kept covered with hot towels, exposing that portion under actual treatment only. Shock will thus be reduced to a minimum. If the intestine is so distended that it can not be replaced within the abdomen, it should be aspirated, if the contents are chiefly gaseous, or incisions may be made which should be closed with Lembert sutures after the contents have been evacuated. The use of chemicals in the abdomen for the purpose of removing infection does not seem justifiable.

As has been stated, patients suffering from septic peritonitis are in a condition of toxemia, more or less profound, which clinically presents all the appearances usually attributed to "shock." It is a matter of common experience that patients in this state bear anesthesia and operations badly. Considerable nicety of judgment is therefore required to determine just how much may be done without adding to the gravity of the already threatening condition. The pulse should be constantly watched, inasmuch as sudden collapse may occur at any moment. Any tendency to failure of the circulation must be met immediately by the administration of full doses of strychnine and digitalis; in fact, it would seem to be good practice to anticipate such collapse by giving the proper dose of these drugs before administering the anesthetic. It is needless to say that evidences of heart-failure would demand a very speedy termination of the operation.

As to the best method of securing drainage of the abdominal cavity, most operators now prefer strips of sterilized gauze.

Sometimes, although the local conditions have been satisfactorily dealt with, the patient sinks rapidly from the fatal dose of the poison circulating in the blood. In such cases the intravenous injection of large quantities of normal salt solution may save life. An instance of this kind has recently been reported by Young, of Baltimore. After an operation upon an appendicular abscess, with evidences of beginning peritonitis, the patient's pulse and temperature gradually rose, and he became more and more prostrated. There were no distinct evidences of peritonitis; the condition appeared to be one of general septicemia. Venesection and saline intravenous injections were decided upon. One and one-half quarts were administered at one time, and two and a half quarts twelve hours later. Improvement followed in each instance, and was especially marked after the last injection. The reporter states that after that there was never any great concern about the boy's welfare. He made an uneventful recovery.—*University Medical Journal.*

